# Sulfur Biogeochemistry of an Oil Sands Composite Tailings Deposit

**DOI:** 10.3389/fmicb.2015.01533

**Published:** 2016-02-03

**Authors:** Lesley A. Warren, Kathryn E. Kendra, Allyson L. Brady, Greg F. Slater

**Affiliations:** School of Geography and Earth Sciences, McMaster UniversityHamilton ON, Canada

**Keywords:** oil sands, composite tailings (CT) deposit, sulfur biogeochemistry, pyrosequencing, PLFA, active bacterial sulfur cycling

## Abstract

Composite tailings (CT), an engineered, alkaline, saline mixture of oil sands tailings (FFT), processed sand and gypsum (CaSO_4_; 1 kg CaSO_4_ per m^3^ FFT) are used as a dry reclamation strategy in the Alberta Oil Sands Region (AOSR). It is estimated that 9.6 × 10^8^ m^3^ of CT are either in, or awaiting emplacement in surface pits within the AOSR, highlighting their potential global importance in sulfur cycling. Here, in the first CT sulfur biogeochemistry investigation, integrated geochemical, pyrosequencing and lipid analyses identified high aqueous concentrations of ∑H_2_S (>300 μM) and highly altered sulfur compounds composition; low cell biomass (3.3 × 10^6^– 6.0 × 10^6^ cells g^−1^) and modest bacterial diversity (H' range between 1.4 and 1.9) across 5 depths spanning 34 m of an *in situ* CT deposit. Pyrosequence results identified a total of 29,719 bacterial 16S rRNA gene sequences, representing 131 OTUs spanning19 phyla including 7 candidate divisions, not reported in oil sands tailings pond studies to date. Legacy FFT common phyla, notably, gamma and beta Proteobacteria, Firmicutes, Actinobacteria, and Chloroflexi were represented. However, overall CT microbial diversity and PLFA values were low relative to other contexts. The identified *known* sulfate/sulfur reducing bacteria constituted at most 2% of the abundance; however, over 90% of the 131 OTUs identified are capable of sulfur metabolism. While PCR biases caution against overinterpretation of pyrosequence surveys, bacterial sequence results identified here, align with phospholipid fatty acid (PLFA) and geochemical results. The highest bacterial diversities were associated with the depth of highest porewater [∑H_2_S] (22–24 m) and joint porewater co-occurrence of Fe^2+^ and ∑H_2_S (6–8 m). Three distinct bacterial community structure depths corresponded to CT porewater regions of (1) shallow evident Fe^(II)^ (<6 m), (2) co-occurring Fe^(II)^ and ∑H_2_S (6–8 m) and (3) extensive ∑H_2_S (6–34 m) (UniFrac). Candidate divisions GNO2, NKB19 and Spam were present only at 6–8 m associated with co-occurring [Fe^(II)^] and [∑H_2_S]. Collectively, results indicate that CT materials are differentiated from other sulfur rich environments by modestly diverse, low abundance, but highly sulfur active and more enigmatic communities (7 candidate divisions present within the 19 phyla identified).

## Introduction

The Athabasca oil sands region (AOSR) in northeastern Alberta, Canada is a globally significant fossil fuel resource. Approximately 1.7–2.5 trillion barrels of bitumen occurs within an area of 75,000 km^2^ (Zhou et al., 2008). The hot water extraction of bitumen from the oil sands results in a fluid tailings waste referred to as fluid fine tailings (FFT) that resist consolidation. In accordance with Alberta's zero discharge policy, all FFT and process-affected water must be stored on site, resulting in large FFT impoundments in the AOSR. It is estimated that the total volume of tailings waste held by all AOSR producers exceeds 700 million m^3^ (Dominski, 2007).

Mining companies are responsible for their waste products, for which the Canadian government has set strict standards regarding tailing pond expansions and reclamation requirements long past mine closure (Bordenave et al., 2010; Dimitriu et al., 2010). Thus, AOSR operators are actively researching wet (i.e., end pit lake) and dry FFT reclamation strategies in order to comply with aggressive government mandated reclamation targets. Dry reclamation involves the use of composite tailings (CT). CT is a mixture of fluid fine tailings (i.e., saline water, suspended Fe^3+^ rich clay minerals, and residual bitumen) and post-processed sand amended with gypsum (CaSO_4_•2H_2_O; 1 kg per m^3^ of FFT; Matthews et al., 2002), which acts as a densifying agent, neutralizing the net negativity of clay minerals to encourage flocculation. The result is a slightly alkaline (pH = 8), moderately saline material with increased trafficability, (Syncrude Canada Ltd., 2010; Ramos-Padrón et al., 2011). Syncrude Canada Ltd., has developed a unique strategy infilling exhausted open-cast mine pits sites with CT and then constructing a dry reclamation surface landscape (i.e., wetland) over top of the CT waste deposit. Current estimates are that 1.2 × 10^8^ m^3^ CT have been created and are currently emplaced in surface pits, while a further 8.4 × 10^8^ m^3^ of tailings await reclamation (World Wildlife Fund, 2010).

Microbial activity within these CT materials is likely. Over the last two decades, studies on oil sands tailings ponds containing FFT have demonstrated a variety of microbial metabolisms including sulfur oxidation and reduction, iron reduction, fermentation, and methanogenesis occur in these systems (Fedorak et al., 2002; Harner et al., 2011; Ramos-Padrón et al., 2011). However, until very recently, investigations have focused dominantly on microbially-mediated methanogenesis (Penner and Foght, 2010; Siddique et al., 2012) in an effort to understand the significant methane fluxes, i.e., 148.6 μM m^−2^ y^−1^ (Small et al., 2014) associated with these systems.

However, in addition to organics such as residual bitumen, solvents such as naphtha and hydrocarbons, oil sands tailings pond waters are also high in ${\text{SO}}_{4}^{2-}$ (Chen et al., 2013). There is emerging recognition that microbial sulfur cycling within oil sands waste contexts is active and may have environmentally important implications associated with H_2_S_g_ production, a toxic, explosive and corrosive risk. Recent research has provided evidence of increasing AOSR sulfur emissions, which suggests generation of H_2_S_g_ within tailings ponds and/or in-filled deposits (i.e., CT; Proemse et al., 2012). Further, well-established communities of sulfate reducing bacteria (SRB) occurring within oil sands tailings ponds have been demonstrated (Gieg et al., 2010; Penner and Foght, 2010; Harner et al., 2011; Ramos-Padrón et al., 2011; Proemse et al., 2012; Chi Fru et al., 2013) supporting the notion that microbial sulfur cycling is active within these contexts. Indeed Chen et al. (2013) demonstrated biologically generated H_2_S_g_ fluxes of 2 × 10^3^ nmol cm^−2^ s^−1^ in experimental FFT microcosm experiments. While Stasik et al. (2014) identified reactive zones of sulfur and iron metabolism occurred directly within the FFT layer of an oil sands tailings pond. Of concern for CT reclamation approaches, Ramos-Padrón et al. (2011) identified the highest rates of sulfate reduction coincided with the highest SRB numbers in situ for an oil sands tailings pond amended with gypsum, added to promote FFT densification and water recycling. Further, the addition of gypsum has been shown to specifically stimulate SRB (i.e., Harner et al., 2011; Chi Fru et al., 2013) as well as inhibit methanogenesis (Ramos-Padrón et al., 2011) suggesting widespread potential for sulfur cycling in oil sands waste residues and especially under sulfate amended conditions such as CT. Indeed, very recent findings (Reid and Warren, 2016) have shown >500 μM ∑H_2_S_(aq)_ and up to 180 ppm H_2_S_(g)_ in CT and overlying sandcap layer porewaters in a CT deposit undergoing pilot wetland reclamation.

The emerging recent genetic surveys of microbial communities associated with oil sands tailings ponds have identified a common bacterial Domain dominance of Proteobacteria, along with higher abundance of Bacteriodetes, Firmicutes and Chloroflexi phyla that possess sulfate- and sulfur-, nitrate-, and iron- reducing, as well as hydrocarbon degrading metabolic capabilities (i.e., Dimitriu et al., 2010; Ramos-Padrón et al., 2011; Yergeau et al., 2012; An et al., 2013a; Chi Fru et al., 2013). As the use of CT as an FFT reclamation strategy is still in the pilot stage, the types and abundances of microbial communities within these materials, as well as the potential for sulfur biogeochemical cycling are currently unknown. Given the substantial sulfate amendment to these materials it is hypothesized that the microbial communities present in CT will share some commonality with those observed in FFT tailings ponds and that microbial sulfur cycling will be active. Thus, the objectives of this study were to characterize depth dependent: (1) bacterial communities via both 454 pyrosequencing and PLFA analyses and (2) sulfur, iron and organic carbon geochemistry in an oil sands CT deposit.

## Materials and methods

### Site description and sampling strategy

Samples were collected from the Kingfisher CT deposit (site location: 57°2′19″ N, 111°34′35″ W) located in the northwest corner of the East-in-Pit tailings deposit at Syncrude Canada Ltd. (Fort McMurray, AB, Canada) in December 2012. Composite tailings deposition into the East-in-Pit began in 2000, thus the deeper CT layers are approximately 12 years older than surface CT materials at the time of collection. An amphibious Fraste ML drill rig fitted with a sonic drill head using an AquaLock Piston Sampler facilitated CT (nonconsolidated material that liquefies upon disturbance) sample collection in ~2 m intervals over 36 m of depth. Based on an initial ∑H_2_S sampling screen (colorimetric, methylene blue assessment and lead acetate paper), five depths were selected for in-depth analyses (2–4, 6–8, 14–16, 22–24, and 32–34 m) that spanned non-detectable to evident/high levels of ∑H_2_S. For each depth sampled, CT cores retrieved as a slurry, were directly extruded into a sterile N_2_-filled anaerobic glove bag, homogenized, physicochemically surveyed (pH, °C, ORP, conductivity; YSI Professional Plus 6-Series Sonde, YSI Incorporated) and subsequently aliquoted for analyses; thus all analyses here represent a bulk characterization over a 2 m depth interval. Samples were collected for: (1) solid-phase Fe and sulfur analysis, total organic carbon/total inorganic carbon (TOC/TIC), and bulk mineralogy; (2) porewater [Fe^(II)^/Fe^(III)^], [∑H_2_S/${\text{SO}}_{4}^{2-}$] and [DOC/DIC] and (3) microbial community and viability analysis (454 pyrosequencing and PLFA). Samples of the drill water used to extrude CT cores were collected and analyzed to account for any microbial and/or geochemical contamination effects.

### Solid phase sample collection and analysis

Samples from each depth were aliquoted into sterile Whirlpak bags (DNA, enrichments, Fe/S analyses, XRD) or C-free glass jars TOC/TIC and PLFA analyses; soaked in 10% HCl for >8 h, rinsed with ultrapure water (18.2 Ω. m cm^−1^, Milli-Q, Millipore) and heated to 450°C for 8 h) and preserved anaerobically in Mylar bags with oxygen absorbing packets (Canadian Survival Company) at −20°C until analysis (4°C for enrichment samples). Samples for Fe/S extractions, XRD and TOC/TIC were air-dried in an anaerobic chamber and finely ground prior to analysis. Given the highly altered nature of the CT materials, in addition to classic mineralogical analyses (XRD), a modified Tessier sequential extraction method was used to assess Fe concentrations of labile sediment components including oxhydroxides and oxides of Fe (Haack and Warren, 2003). Fe concentrations associated with each sequential extraction step were determined in triplicate using the Ferrover HACH method (Ultraspec 2000, UV/visible spectrophotometer, Pharmacia Biotech, Cambridge, UK). Background contamination was accounted for through procedural blanks and was found to be negligible (<5%). Total sediment Fe concentrations were calculated through the sum of Fe concentrations across the six fractions while the proportion of bioavailable Fe^(III)^ (e.g., Fe^(III)^ available to iron reducing bacteria, IRB) was approximated through the sum of Fe concentrations in the easily reducible and reducible fractions. Solid-phase sulfur components were quantified through acid volatile sulfide analysis (AVS, i.e., reduced sulfide phases) and acid extractable sulfate (AES) analysis using a method adapted from Burton et al. (2007) and Hsieh et al. (2002). Acid-associated sulfide and sulfate concentrations were determined colorimetrically using the Sulfide and SulfaVer 4 HACH methods respectively (Ultraspec 2000). Sediment C contents [total organic C (TOC)/total inorganic C (TIC)] were analyzed on a Shimadzu TOC-L Analyzer with a Solid Sample Combustion Unit (Mandel Scientific) using the 680°C combustion catalytic oxidation method (Shimadzu Corporation, 2013). CT bulk mineralogy was determined by X-ray diffractometry (XRD), using a high resolution Bruker D8 Advance Powder Diffractometer with a germanium monochormator in conjunction with DFFRAC PLUS Evaluation software at the McMaster University X-Ray Diffraction Facility (Brockhouse Institute for Materials Research, McMaster University, Hamilton, Ontario).

### Analysis of CT porewaters

CT has a high “fines” (clay) content (~18%), which precludes rapid field collection of redox sensitive dissolved (<0.2 μm or even 0.45 μm) porewater samples through either filtration or settling. Thus, Slide-a-Lyzer cassettes (Thermo Scientific, 3 mL volume, dialysis membranes 20 kD molecular weight cut-off) were used. CT was extruded directly within an Atmosbag glove bag under N_2_ gas, aliquoted into Whirlpak bags containing Slide-a-Lyzers pre-filled with degassed ultrapure water (3 Slide-a-Lyzers per 2 L bag of CT), and preserved anaerobically at 4°C for 4 days (experimentally determined equilibration time for a known ${\text{SO}}_{4}^{2-}$ concentration spike within a sand porewaters using the Slide-a-Lyzers). Samples were anaerobically extracted from the Slide-a-Lyzers and analyzed for [∑H_2_S], [${\text{SO}}_{4}^{2-}$], [Fe^(II)^], [Fe^(III)^] (quantified using reagents and manufacturer supplied protocols from Hach Company with Ultraspec 2000 spectrophotometer) and DOC/DIC content. Samples for DOC/DIC analysis were filtered (0.7 μm) into C-clean glass vials and frozen at −20°C until analysis (<28 days). DOC/DIC concentrations were measured on a Shimadzu TOC-L analyzer (Mandel Scientific) and procedural blanks were analyzed to account for background C contamination (DOC < 25%, DIC < 1%).

### DNA extraction and analysis of pyrosequencing data

Total community DNA was extracted from CT sediment samples and drill water (to assess any contamination) using the PowerSoils™ DNA Isolation Kit (Mo Bio Laboratories, Carlsbad, CA, USA) using low biomass modifications. Samples were submitted for 454 pyrosequencing to MR DNA Next Generation Sequencing and Bioinformatics Services (Shallowater, TX, USA). Polymerase chain reaction (PCR) was performed using the universal Eubacterial primers 27F (5′AGRGTTTGATCMTGGCTCAG-3′) and 530R (5′-CCGCNGCNGCTGGCAC-3′) and Archaeal primers 344F (5′-ACGGGGYGCAGCAGG CGCGA-3′) and 915R (5′-GTGCTCCCCCGCCAATTCCT-3′ in which samples were subjected to the following conditions: 28 cycles of 94°C (30 s), 53°C (40 s), and 72°C (60 s) with a final elongation step at 72°C (300 s). Sequencing was performed using the Roche 454 FLX genome sequencer system with FLX Titanium reagents (Roche Applied Sciences, IN, USA) as previously described (Dowd et al., 2008; Wolcott et al., 2009). Data was processed using a proprietary analysis pipeline where data was depleted of barcodes and primers, sequences were denoised, chimeras were removed and operational taxonomic units (OTUs) were generated (binned at 97% similarity). Sequences were classified using BLASTn, compared against a complied GreenGenes database and analyzed for community composition. These sequence data have been submitted to the GenBank database under SRA accession No. SRP066063 (release date 2016-02-15). Drill water sequences were removed from CT sequences at the genus level to account for drill water contamination during field sampling. Sequences were aligned using the Ribosomal Database Project Pyrosequencing Alignment tool and a phylogenetic tree was generated using FastTree (Cole et al., 2009; Price et al., 2009).

### PLFA analyses

Approximately 70 g of lyophilized CT was extracted from each sample using a modified Bligh and Dyer (1959) method and purified using silica gel chromatography to separate lipids into non-polar, neutral and polar fractions. Polar phospholipids were converted to fatty acid methyl esters (FAMEs) via mild alkaline methanolysis (Guckert et al., 1985). FAMEs were separated using gas chromatography-mass spectrometry (GC-MS) with an Agilent GC-MS instrument (Agilent Technologies Inc., Santa Clara, California, USA) equipped with DB-XLB column (30 m × 0.32 mm i.d. × 0.25 μm film thickness) using a temperature program of 50°C (1 min.), 20°C/min to 130°C, 4°C/min to 160°C, 8°C/min to 300°C (5 min). PLFA assignment was based on retention time and mass spectra of standards (Bacterial Acid Methyl Esters Mix, Matreya Inc., Pleasant Gap, Pennsylvania, USA). PLFAs are named as follows; number of carbons: number of double bonds, followed by position (Δ) of the double bond from the carboxyl group. *Iso*- and *anteiso*- are denoted by i or a respectively. Methyl branching is indicated as the position of the Me from the carboxylic group. Br indicates a branch at an unknown location followed by the total number of carbons. Cy indicates cyclopropyl. PLFA concentrations (μg/g dry weight) are used as a proxy for microbial biomass abundance. Viable cell estimates from PLFA data were generated using a generic conversion factor of 2.0 × 10^4^ cells/pmol PLFA (Green and Scow, 2000). Picolinyl ester derivatives used to identify double-bond and methyl-branch positions were prepared as in Dubois et al. (2006).

### Statistical analyses

All values are reported as means (*n* = 3) plus or minus one standard error. Error bars on all graphs represent one standard deviation. Depth dependent trends were assessed using One-way ANOVA tests while two-tailed correlations were used to compare UniFrac PCA and environmental variables (IBM SPSS Statistic 21 software package). Trends were considered significant at α = 0.05, unless otherwise noted. The Unifrac web application (Lozupone and Knight, 2005; Lozupone et al., 2006) was used to evaluate microbial community structure with depth through significance tests and principal component analysis (PCA). Principal components were generated based on the pyrosequence data and were each correlated against geochemical parameters including temperature, pH, ORP, conductivity, concentrations of porewater species (∑H_2_S, ${\text{SO}}_{4}^{2-}$, Fe^2+^, Fe^3+^, and DOC/DIC) and solid phase constituents (total Fe, bioavailable Fe, AVS/AES, and TOC).

## Results

### CT geochemistry

**Figure 1:**
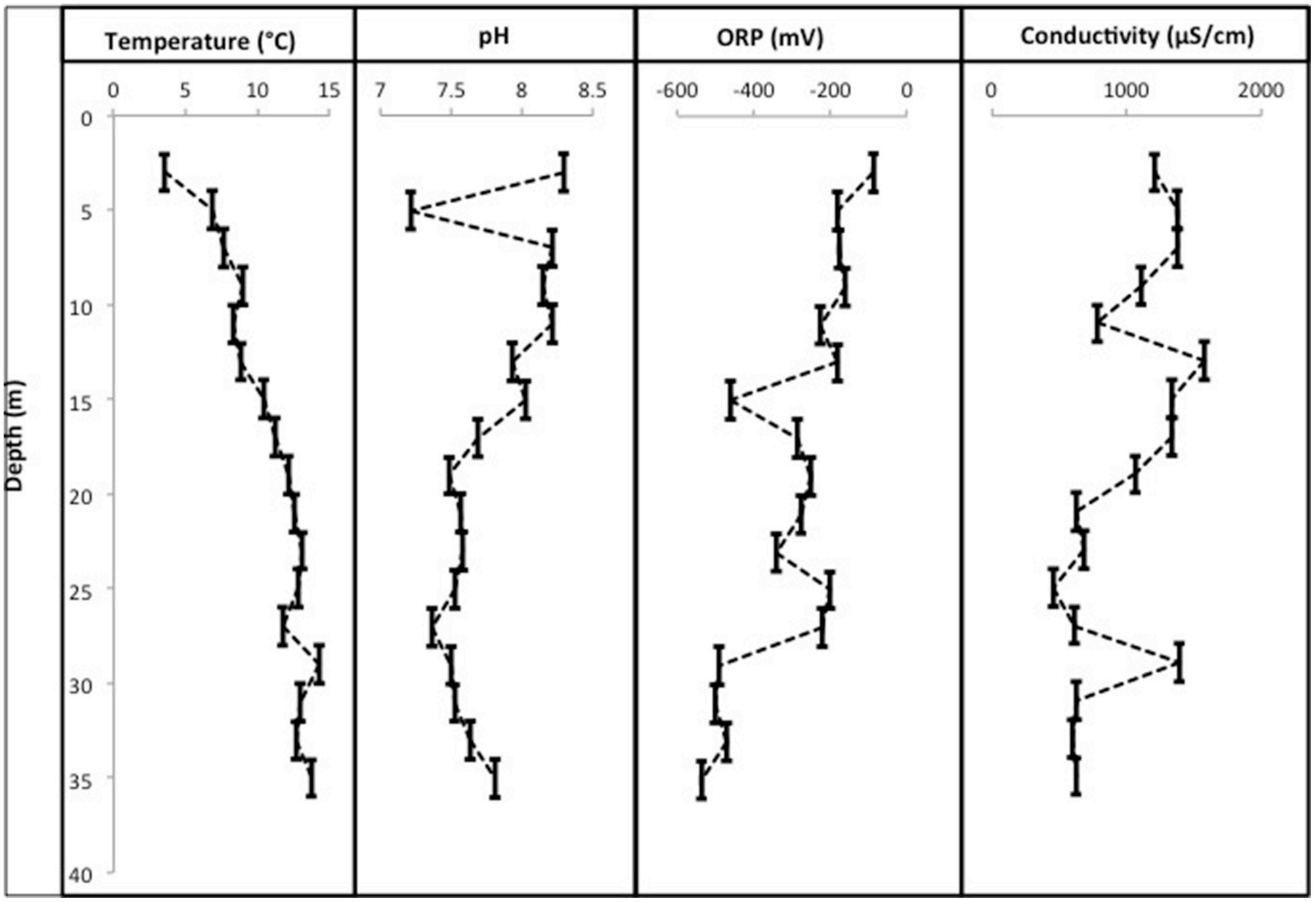
**Physicochemical profiles for pH, °C, conductivity, and ORP over 36 m within the CT deposit**. Sampling points represent a composite average for each of the 2 m sampling depths (liquefaction of CT upon core retrieval generates a nonconsolidated slurry sample).

**Figure 2:**
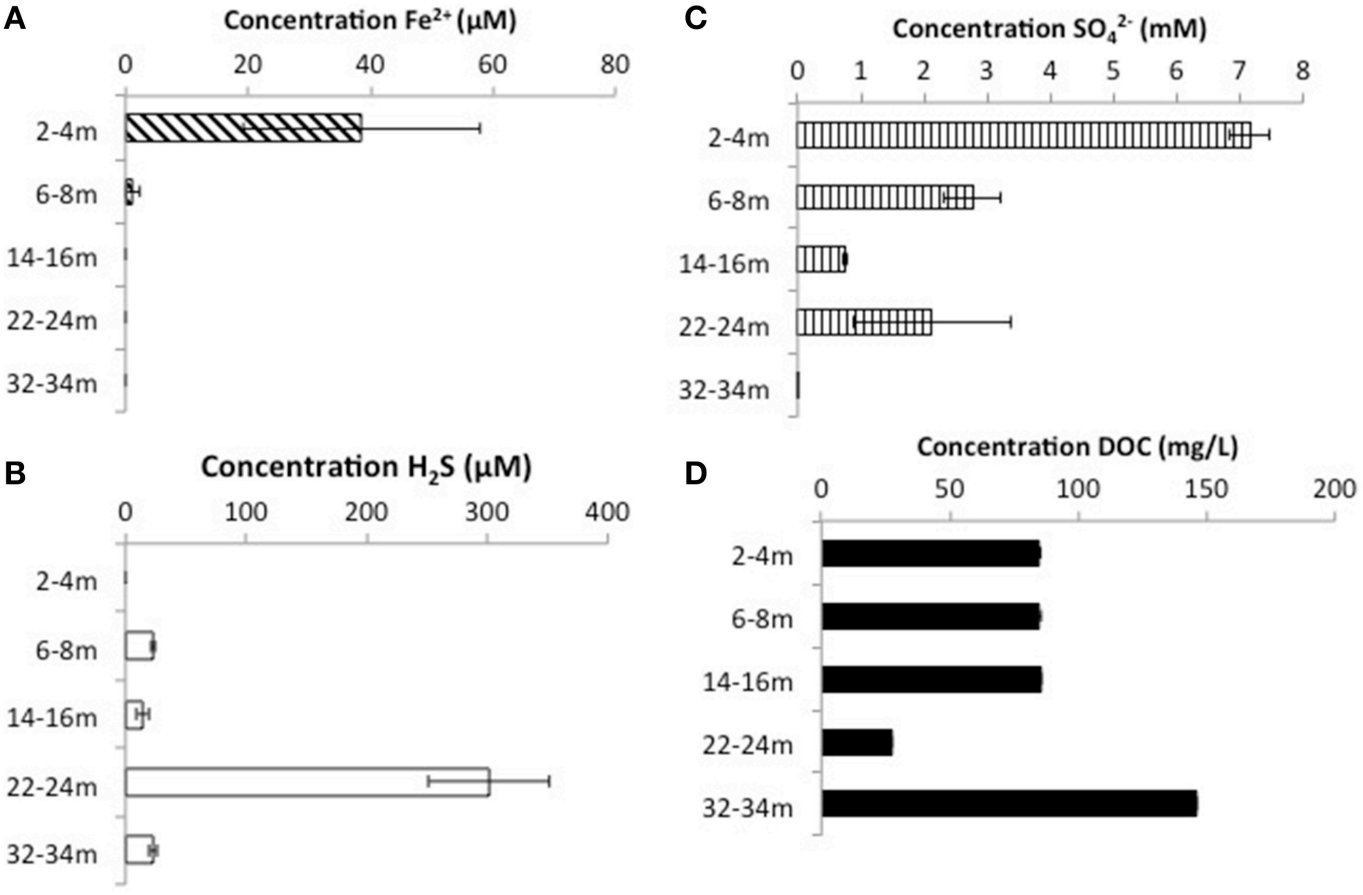
**Porewater concentrations of (A) ∑H_2_S, (B) Fe^2+^, (C) ${\text{SO}}_{4}^{2-}$, and (D) DOC with depth throughout the CT deposit**. Values displayed are means generated from triplicate analyses with error bars representing one standard deviation of variance.

CT porewaters were moderately saline (~460–1390 μS/cm), highly reducing (ORP −90 to −538 mV) with a circumneutral pH (~7.2–8.3) and temperature increasing with depth (3.5–14.3°C) (Figure 1). Solids analyses identified 0.75–1.22% (w/w) TOC across sampled depths, a depth-dependent increasing presence of Fe(III) minerals, no detectable S-bearing minerals (XRD Supplementary Information, Table S1), and low AVS and AES (0.4–0.5 μmol/g and ~0.01 μmol/g respectively) concentrations (Table 1). Three distinct sulfur and Fe redox zones are evident in CT porewater profiles over the five sampling depths from 2 to 34 m (Figure 2). A restricted iron reducing surficial zone limited to the two surficial sampling depths (2–4 and 6–8 m). Fe^(II)^ was detected at concentrations of 38.5 μM and 1.2 μM respectively at these two depths. An IRB-SRB transition zone at 6-8 m as evidenced by the detection of both Fe^(II)^ and ∑H_2_S for this sample. Below this 6–8 m IRB-SRB transition zone, appears to be an extensive SRB zone including a maximal ∑H_2_S level at 22–24 m depth (301.5 μM), twelve times higher than any other depth (~14–23 μM; *p* < 0.01; Figure 2).

**Table 1:** **Depth dependent solid phase S, Fe, and C analyses reporting mean values with one standard deviation**.

**Depth (m)**	**Bioavailable Fe (μmol/g)**		**Total Fe (μmol/g)**		**OrgC [%)**		**AVS (μmol/g)**		**AES μmol/g)**	
	**Mean**	±	**Mean**	±	**Mean**	±	**Mean**	±	**Mean**	±
2–4	22.58	0.45	35.89	0.24	0.79	0.16	0.43	0.00	0.01	0.00
6–8	25.79	0.43	39.67	1.25	0.75	0.09	0.53	0.00	0.01	0.00
14–16	27.73	1.41	41.26	0.64	0.87	0.12	0.51	0.01	0.01	0.00
22–24	38.18	0.40	62.30	0.57	0.94	0.04	0.49	0.04	0.01	0.00
32–34	47.24	0.93	73.39	1.05	1.22	0.01	0.43	0.01	0.01	0.00

*Means were generated based on triplicate analyses with one standard deviation (SD) of variation. Bioaccessible Fe [i.e., Fe^(III)^] for IRB was quantified as the Σ of sequential extraction steps 2 and 3 (amorphous and crystalline Fe oxides, Haack and Warren, 2003), Total Fe, Σ of Fe extracted in all 6 extraction steps*.

**Figure 3:**
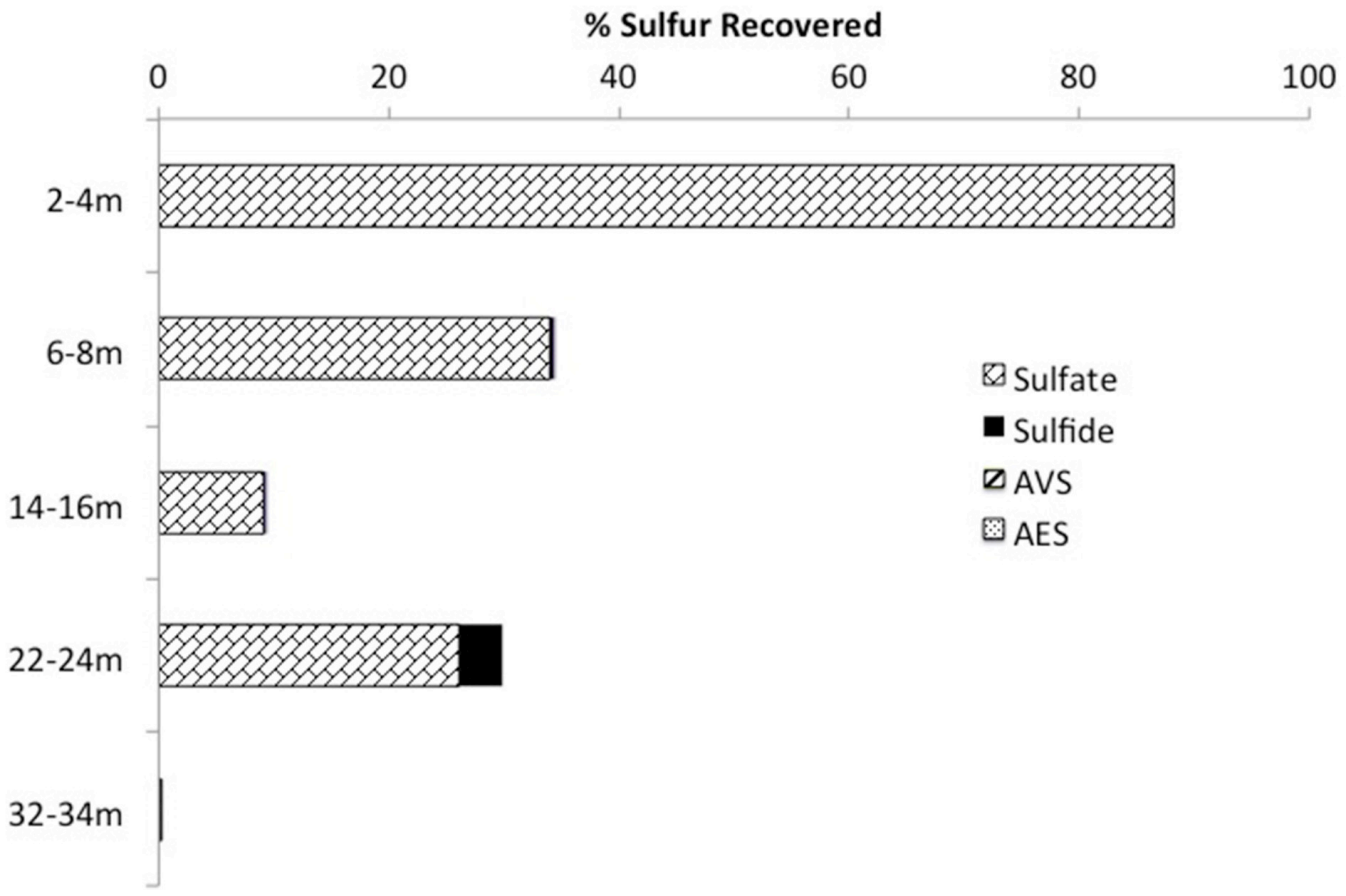
**Sulfur mass balance with depth in the CT deposit including the summation of aqueous (e.g., ${\text{SO}}_{4}^{2-}$, ∑H_2_S) and solid (e.g., AVS, AES) forms of sulfur (n.b. no S minerals detected by XRD analyses and >99% of all S measured as porewater ${\text{SO}}_{4}^{2-}$)**.

**Figure 4:**
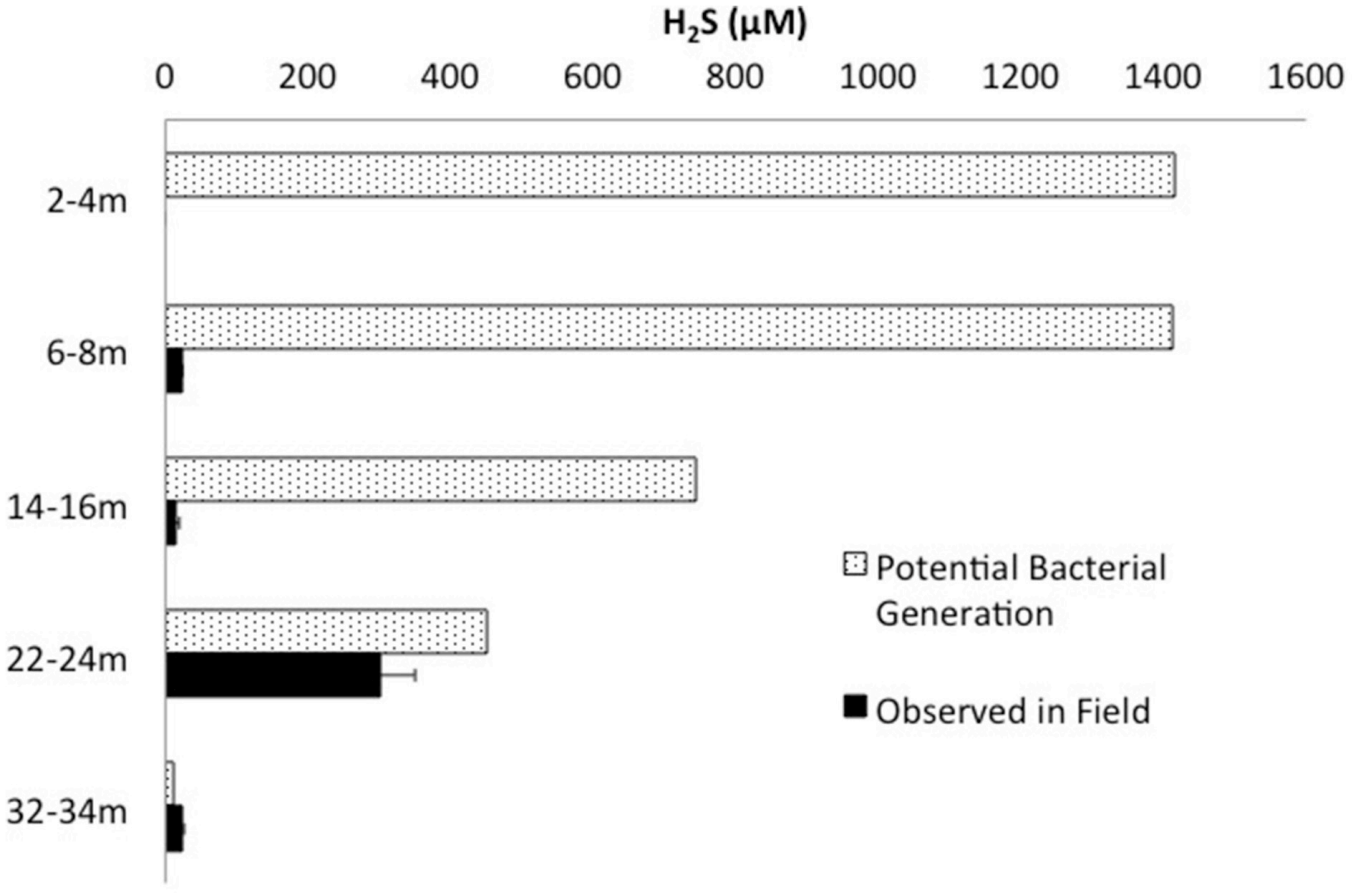
**∑H_2_S concentrations measured in the field plotted against the theoretical maximum ∑H_2_S possible by bacterial sulfate reduction based on measured concentrations of ${\text{SO}}_{4}^{2-}$ and DOC (and assuming all DOC = CH_2_O)**.

Mass balance sulfur calculations, summing all detected sulfur (e.g., porewater [${\text{SO}}_{4}^{2-}$] (>99% of total sulfur recovered for all depths) and [∑H_2_S], and associated solid phase AVS/AES concentrations, (n.b. no detectable sulfur containing minerals by XRD, Supplemental Information Table S1), identified an average loss of ~70% of the ${\text{SO}}_{4}^{2-}$ added through gypsum amendment to CT and the highest loss of 99% at the deepest depth (Figure 3). Relative SRB activity, calculated as the ratio of observed porewater ∑H_2_S values to the theoretical maximum possible ∑H_2_S based on DOC (all DOC assumed to be CH_2_O) and ${\text{SO}}_{4}^{2-}$ concentrations at each depth (Equation 1), identified highest evident SRB activity at the two deepest depths sampled (67 and 230% respectively, Figure 4) relative to much lower values at the shallower depths (<2%).

(1) $$\begin{array}{r} {\text{SO}}_{4}^{2-}+2{\text{CH}}_{2}\text{O}\to {\text{H}}_{2}\text{S}+2{\text{HCO}}_{3}^{-} \end{array}$$

### Bacterial community composition, diversity, and abundance

Four hundred fifty four sequencing failed to amplify archaeal sequences from these CT samples and thus results of the pyrosequence genetic survey are limited to the bacterial domain. Further, since our survey is PCR-based, it is subject to biases from the PCR process itself or from the hypervariable regions selected for amplification (An et al., 2013b), and thus results should not be overinterpreted. However, literature evidence of: (1) active bacterial sulfur metabolism within FFT (e.g., Chen et al., 2013; Stasik et al., 2014); (2) bacteria involved in sulfur cycling tend to outcompete methanogens under sulfate amended conditions (Ramos-Padrón et al., 2011; Chi Fru et al., 2013); and (3) pyrosequencing and metagenomic sequencing identifying the same major taxa (An et al., 2013a) collectively lend support to the opportunity to gain valid insights from these data with respect to bacterial domain CT sulfur metabolism. In particular, as summarized below, the agreement of our genetic survey results for bacterial taxa with the known literature on oil sands tailings ponds and depth dependent alignment of the bacterial community structural results with the geochemical results lends further credence to the utility of the pyrosequencing survey results presented here.

**Figure 5:**
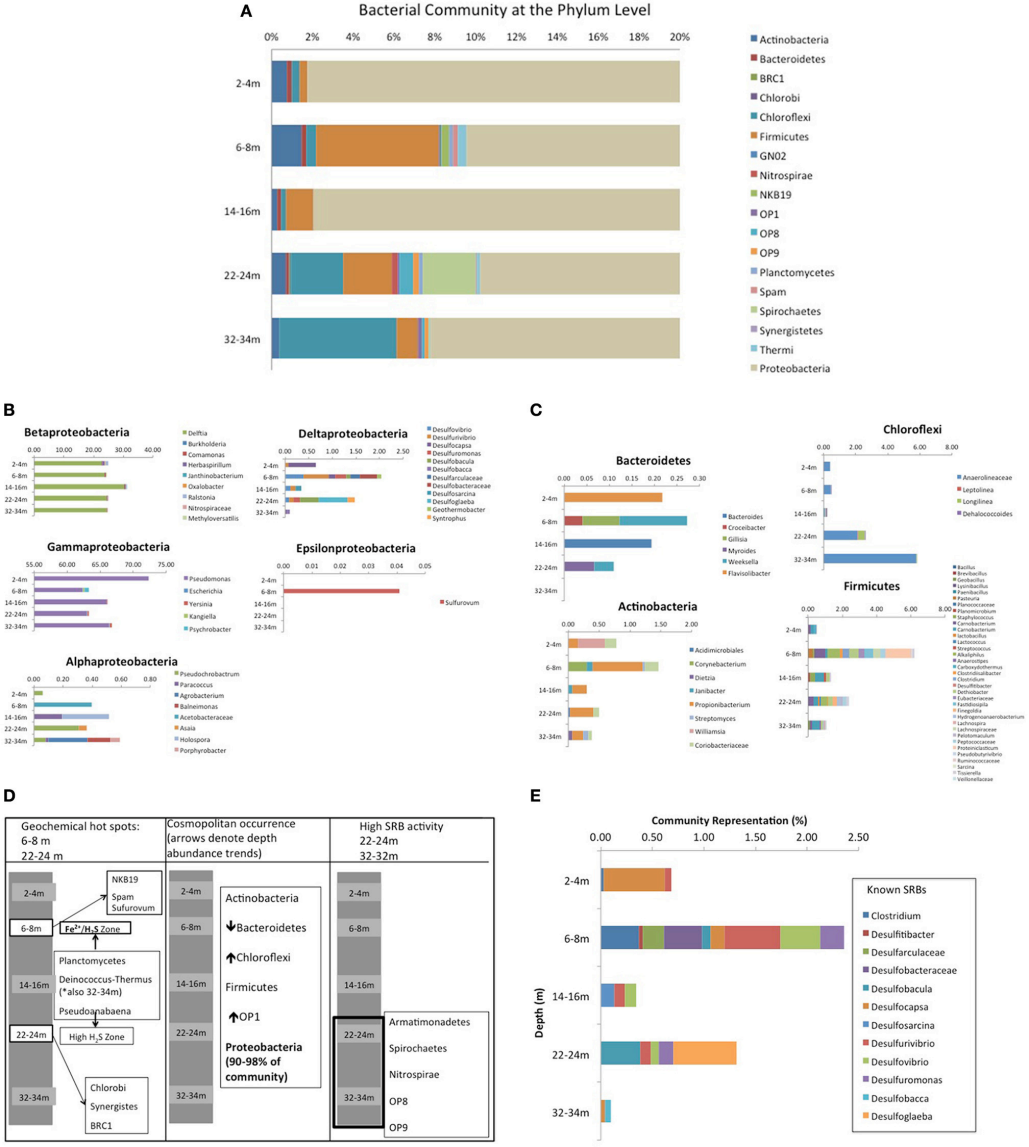
**(A)** CT bacterial community diversity with depth, organized at the Phylum level. Due to the dominance of Proteobacteria (90–98% of total community), the x-axis has been magnified to represent 0–20% of community diversity (where anything beyond 14% is Proteobacteria) to highlight the other contributing Phyla. **(B)** Depth dependent Proteobacterial class trends for the 5 sampled depths in the CT deposit. **(C)** Non Proteobacteria cosmopolitan Phyla depth dependent trends for the 5 sampled depths in the CT deposit. **(D)** Non cosmopolitan Phyla depth dependent trends for the 5 sampled depths in the CT deposit. **(E)** Depth dependent trends of known SRB for the 5 sampled depths in the CT deposit.

Roche 454 pyrosequencing of the five depth samples yielded a total of 29,719 bacterial 16S rRNA gene sequences, which clustered into 131 OTU's with an average read length of 463 base pairs, representing 30 classes within 19 phyla, including 7 candidate divisions (37%) (Figure 5A) [n.b., all sequences associated with drill water (i.e., unavoidable contamination) were removed from analyses using the following approach: any sequences only present or present at higher abundance in drill water were deleted or subtracted from CT samples if present in drill water at lower abundance; Supplementary Information Table S2]. Proteobacteria dominated (>90%) all communities (Figure 5A) with the 5 classes diverging in depth dependent trends (Figure 5B). δ and γ classes showed respectively highest and lowest abundance at 6–8 and 22–24 m geochemical hotspots (Figure 3), while β proteobacteria dominated by *Delftia* spp., showed the highest abundance at 14–16 m (low sulfur activity depth), α Proteobacteria showed the lowest abundance at the shallow depth where only Fe(II) occurred and higher abundance at underlying depths where ∑H_2_S occurred, and ϵ Proteobacteria (*Sulfurovum)* only detected at 6–8 m (IRB-SRB transition). Other ubiquitous phyla identified included Firmicutes, Actinobacteria, Chloroflexi, and Bacteriodetes, but at lower abundances (<5% of all sequences) and displaying differing depth dependent trends (Figure 5C). Seven candidate divisions were identified to occur across the 5 depths (OP1, OP8, OP9, GN02, NKB19, Spam, and BRC1).

Depth dependent trends emerged for some phyla that map to observed geochemical zones (Figure 5D). Candidate divisions *GNO2, NKB19*, and *Spam* were only found at 6–8 m (co-occurring porewater Fe^(II)^ and ∑H_2_S, hypothesized to reflect an IRB-SRB transition zone; Figure 2), while Synergistetes, Chlorobi, Nitrospirae, and candidate division BRC1 were only detected at 22–24 m where the highest ∑H_2_S concentration was detected. Planctomycetes and Pseudoanabaena occurred at both the IRB-SRB transition 6–8 m zone and the high [∑H_2_S] depth of 22–24 m, while Armatimonadetes, Spirochaetes, Nitrospirae, OP8, and OP9 occurred exclusively at the deepest depths sampled (22–24 and 32–34 m) where the lowest [${\text{SO}}_{4}^{2-}$] and % sulfur recovery occurred, consistent with greater SRB activity (Figures 2–5).

Delta proteobacteria, containing the majority of known SRB, accounted for only 0.1–2% of the overall community, however their highest representation and greatest diversity occurred at 6–8 m (2%; overlapping Fe^(II)^/∑H_2_S porewater zone, Figure 2) and 22–24 m (1%; depth of highest porewater [∑H_2_S]) (Figure 5E). Across all depths, ten genera of known sulfate and sulfur reducing bacteria (SRB) were identified including *Desulfovibrio* spp., *Desulfuromonas* spp., *Desulfocapsa* spp., and *Clostridium* spp.

Shannon-Weiner index values of bacterial diversity (H'; Table 2) ranged between 1.4 and 1.9, where the highest bacterial diversity was found at 6–8 and 22–24 m; namely the two geochemical hotspot depths of overlapping Fe^(II)^/∑H_2_S (i.e., IRB-SRB transition zone) and the depth of highest observed porewater [∑H_2_S] (Table 2, Figure 2). The lowest bacterial diversity (5 Phyla) occurred at the surface 2–4 m depth (detectable Fe^(II)^, no detectable ∑H_2_S; Figures 2, 5).

**Table 2:** **Shannon-Weiner diversity index, species richness (s), and evenness values for CT microbial communities with depth**.

**Depth (m)**	**Alpha diversity**		
	**Species richness (s)**	**Shannon index (H')**	**Evenness**
2–4	33	1.363	0.39
6–8	74	1.914	0.44
14–16	39	1.415	0.39
22–24	74	1.772	0.41
32–34	46	1.510	0.39

**Figure 6:**
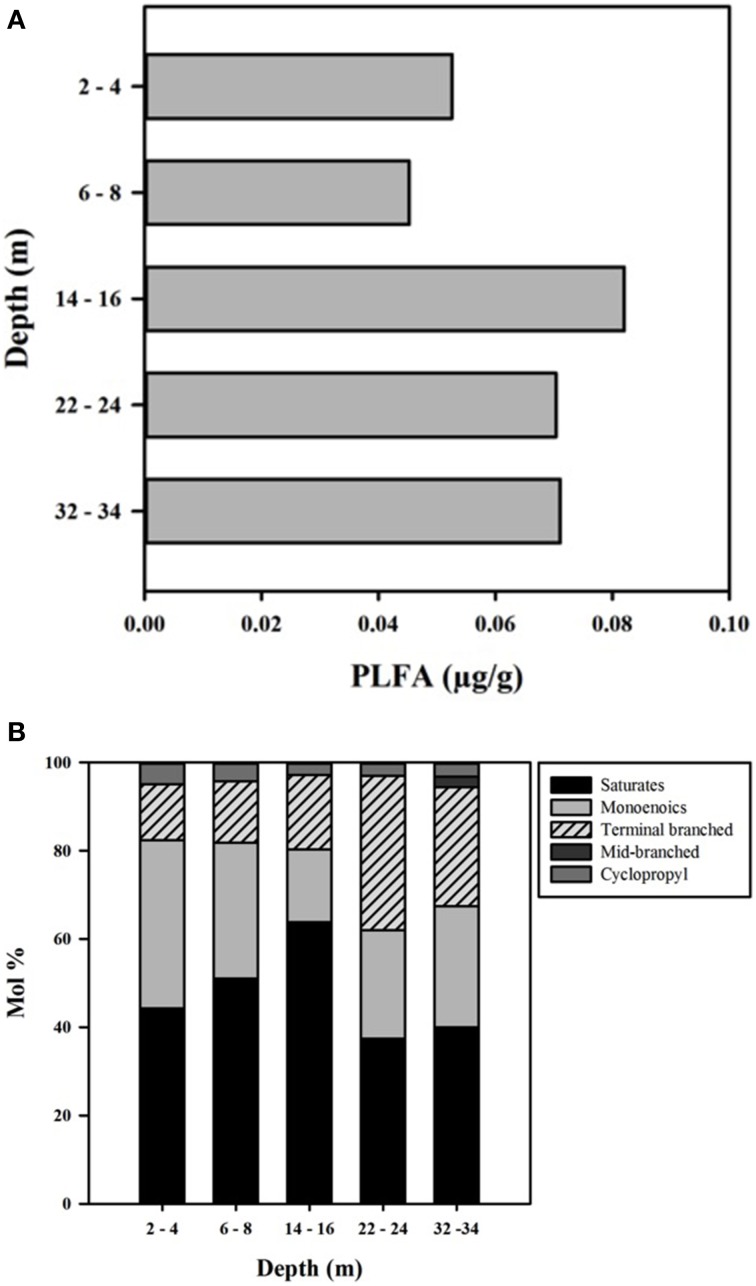
**(A)** PLFA concentration (μg/g) with depth in the CT deposit. **(B)** Profiles of individual PLFA expressed as mol % with depth in the CT deposit.

PLFA concentrations ranged from 45 to 82 μg/g, with the highest value observed at 14–16 m (Figure 6). The total amount of PLFA corresponded to an estimated cell density range of 3.3 × 10^6^– 6.0 × 10^6^ cells g^−1^ (Figure 6A). Overall, the deeper core samples (14–16, 22–24, and 32–34 m) had higher biomass relative to shallow CT samples; however the number of samples was too small to determine the statistical significance of this trend.

PLFA ranged from 14:0 to 20:0 and distributions were dominated by saturated and mono-unsaturated PLFA (mono-unsaturated predominantly 16:1Δ11 and 18:1Δ11), which comprised 80–82% of the distribution at the three shallower depths and 60–68% at the two deepest depths (Figure 6B). The decreased proportion of these PLFA at depth was due to increased presence of terminally branched PLFA (*iso*- and *anteiso*- isomers of C_15_, C_16_, and C_17_), 13–17% in the shallower samples, 26–35% in the deepest two samples, with higher proportions of the longer chain isomers present at depth. The cyclopropyl PLFA cy17:0, indicative of gram-negative bacteria (Parkes and Taylor, 1983) or as a response to environmental stress including nutrient depravation (Kieft et al., 1994; Petersen and Klug, 1994) decreased from 5% in the upper two samples to ~3% in the deeper three samples.

### Geochemical drivers of microbial community structure

**Figure 7:**
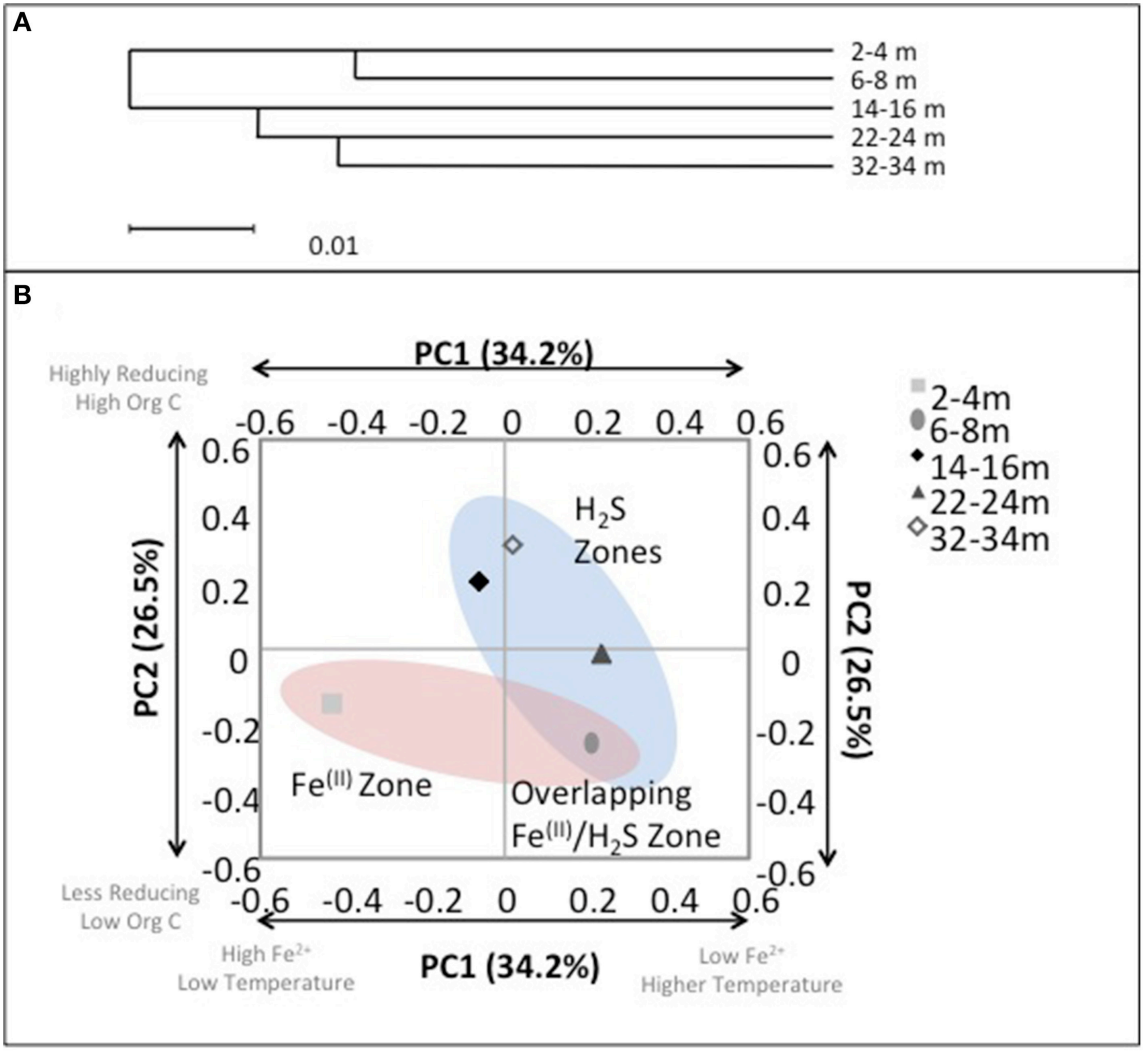
**(A)** UniFrac cluster analysis illustrating the similarities between bacterial communities with depth. **(B)** Microbial community composition with depth in CT deposit as a function of principal components 1 (PC1) and 2 (PC2). Axis labels refer to the principal components and the total percentage of variance explained by each component. Text boxes identify correlated environmental characteristics associated with microbial community composition: ORP and conductivity (weakly negatively correlated with PC1, *p* < 0.1) and organic C (negatively correlated with PC2, *p* < 0.05). Highlighted clusters correspond with zones of observed porewater [∑H_2_S] and [Fe^2+^].

Despite the age of the deposit being relatively young, i.e., CT deposition into the East-In-Pit only began in 2000, ~12 years before this sampling campaign; evident depth dependent microbial community structure variation emerged that was consistent with geochemical zonation (Figure 7). Hierarchical cluster analysis of the microbial communities using the UPGMA (Unweighted Pair Group Method with Arithmetic Mean) algorithm identified that surficial microbial communities (2–4 and 6–8 m) were closely similar and distinct from those occurring at the three deeper depths where no Fe^(II)^ was detected (14–16, 22–24, and 32–34 m; *p* < 0.01) (Figure 7A) suggesting a discernible difference between Fe^(III)^ reducing and ${\text{SO}}_{4}^{2-}$ reducing communities. Principal coordinates analysis (PCA) based on an environmental distance matrix indicated that communities from the four deeper depths with detectable porewater ∑H_2_S concentrations, i.e., including co-occurrence of Fe^(II)^ and ∑H_2_S at 6–8 m, clustered together and separately from the most shallow depth where only detectable porewater Fe^(II)^ was observed (Figure 7B). Principal component (PC) PC1 and PC2 axes explained over 60% of community variation. PC1 was negatively correlated with porewater [Fe^(II)^] (*R*^2^ = 0.8) and moderately positively correlated with temperature (*R*^2^ = 0.5) while PC2 was negatively correlated with ORP (*R*^2^ = 0.8) and positively correlated with TOC (*R*^2^ = 0.7). Hence, community structuring revealed two distinct CT zones; (1) a shallow zone characterized by detectable [Fe^(II)^], lower temperatures, lower TOC concentrations and less reducing conditions (<8 m); and (2) a deeper more extensive zone (14–34 m) characterized by no detectable porewater Fe^(II)^, higher temperatures, greater TOC concentrations and more reducing conditions. These analyses clearly identified the transition depth of 6–8 m, where IRB activity was transitioning to SRB activity as intermediary to these two zones (Figure 7B).

## Discussion

### CT bacterial community diversity, structure, and abundance

Consistent with characteristics of extreme contexts (Reysenbach and Hamamura, 2008), 454 pyrosequencing and PLFA analyses indicated CT hosts limited bacterial diversity and biomass (Table 2, Figure 7). 131 OTUs were identified spanning 19 phyla, including 7 candidate divisions (Figure 5), which translated into considerably lower Shannon Weiner Index, H' values, 1.3–1.9 (Table 2) compared to typical soil values (i.e., >3, Li et al., 2014a). Further, CT PLFA results exhibited low bacterial cells counts (Figure 6A), ~1–2 orders of magnitude lower compared to other petroleum rich systems such as oil reservoirs (Hallmann et al., 2008), contaminated salt marshes (Mahmoudi et al., 2013) and tailings ponds (Ahad and Pakdel, 2013) consistent with a highly geochemically restrictive habitat. Further, the microbial biomass as measured by PLFA, was relatively low (Figure 6A). Microbial biomass as reflected in PLFA concentrations was greatest at depths 14 m and below, i.e., the oldest materials with the highest alteration of sulfur components (Figures 3, 4). PLFA cell density estimates of 3.3 × 10^6^– 6.0 × 10^6^ cells g^−1^ (Figure 6A) are 1–2 orders of magnitude lower compared to other petroleum rich systems such as oil reservoirs (Hallmann et al., 2008), contaminated salt marshes (Mahmoudi et al., 2013) and tailings ponds (Ahad and Pakdel, 2013). These results suggest that CT is not as hospitable as the original tailings ponds from which the FFT was sourced, resulting in a focused refinement of the microbial communities that establish in CT from the original FFT parent communities.

Proteobacteria, particularly γ (62–71%; *Pseudomonas*) and β (24–31%, *Delftia*) classes, dominated communities at every depth (Figure 5). This ubiquitous phyla is metabolically diverse, capable of surviving in extreme environments and able to use a variety of carbon compounds (Islam et al., 2014). The abundance of Proteobacteria within oil sands tailings ponds has been previously identified (Yergeau et al., 2012; An et al., 2013a; Chi Fru et al., 2013) and specifically identified Beta proteobacteria and Gamma proteobacteria domination in their depth dependent pyrosequence and metagenomic sequencing assessment of a tailings pond. These results identify that the FFT incorporated into CT are a legacy source of microorganisms capable of continued existence within the CT matrix. The dominant bacterial phyla identified in this pyrosequencing survey other than Proteobacteria include Firmicutes, Actinobacteria, Chloroflexi, and Bacteriodetes (Figure 5), again highly consistent with important community members identified in oil sands tailings pond and gypsum amended tailings pond surveys (i.e., Ramos-Padrón et al., 2011; Yergeau et al., 2012; An et al., 2013a,b).

Many bacterial strains affiliated with the Proteobacteria phylum identified here (e.g., *Pseudomonas, Comamonas, Rhodobacter, Syntrophus*, and *Rhodocyclus*) have been found in association with hydrocarbon biodegradation and may assist the breakdown of complex molecules (Wang et al., 2013; Das and Kazy, 2014). An et al. (2013b) identified Proteobacteria, Firmicutes, and Actinobacteria to be involved in upper pathways of hydrocarbon breakdown, while Delta proteobacteria were involved in lower pathway hydrocarbon degradation using 454 pyrosequencing and metagenomic sequencing of oil sands tailings pond depth dependent microbial communities. Branched cyclopropyl, and odd-numbered straight chained, saturated PLFA samples found at all depths here (Figure 6B), have also been observed in association with other hydrocarbon rich environments (e.g., Aries et al., 2001; Hallmann et al., 2008), suggesting biodegradation of CT components. Terminally-branched *iso*- and *anteiso*- PLFAs present at all depths are common in Gram-positive bacteria and some anaerobic Gram-negative bacteria (e.g., some SRB; Boon et al., 1977; Taylor and Parkes, 1983; Londry et al., 2004). Gram-positive fermentative bacteria such as members of the *Firmicutes* phylum have been suggested to play a role in degrading recalcitrant compounds (e.g., Röling et al., 2003).

Ten genera of bacteria with *known* sulfate/sulfur reducing metabolism capabilities were identified across the 5 depths, accounting for less than 2% of the total bacterial community (Figure 5E) although up to 90% of identified OTUs possess putative SRB capabilities. Many of the identified genera have been reported as community members in other anaerobic, hydrocarbon associated environments. *Desulfarculacea*, Desulfobacteraceae, *Desulfosarcina, Desulfovibrio, Desulfuromonas, Desulfobacca*, and *Desulfoglaeba* are known sulfate reducers (Celis et al., 2013; Montoya et al., 2013; Kleindienst et al., 2014; Mand et al., 2014; Pedersen et al., 2014; Piceno et al., 2014; Laban et al., 2015). *Clostridium* (Sallam and Steinbüchel, 2009) has been associated with thiosulfate (S_2_${\text{O}}_{3}^{2-}$) and elemental sulfur (S^0^) reduction, while *Desulfitibacter* has been shown to be unable to reduce ${\text{SO}}_{4}^{2-}$ but is involved in sulphite (${\text{SO}}_{3}^{2-}$) reduction and *Desulfocapsa* has been demonstrated to carry out sulfur disproportionation (Ramos-Padrón et al., 2011). Interestingly, Desulfobacteraceae have been shown to be able to degrade naphthenic acids, a key residual component of FFT (Laban et al., 2015), and *Desulfosarcina* and *Desulfuromonas* have been identified as capable of hydrocarbon degradation (Piceno et al., 2014). *Desulfobacca* have been shown to be acetotrophic (Celis et al., 2013; Montoya et al., 2013), while *Desulfoglaeba* has been identified as alkane degraders (Callaghan et al., 2010). Thus, there appears to be a well-adapted suite of SRB capable of using available carbon sources within the CT matrix.

The highest diversity of *known* sulfur reducing bacteria (sulfate/sulfur-reducing; SRB) occurred at the two geochemical hotspots of IRB-SRB transition at 6 – 8 m and high ∑H_2_S generation (22–24 m; Figures 2, 5E). PLFA analyses identified the presence of viable SRB through the detection of 10me16:0, considered a biomarker for the genus *Desulfobacter* (Taylor and Parkes, 1983; Coleman et al., 1993) at all depths. Notably this PLFA was only at sufficient concentration for quantification at 32 – 34 m, where the lowest observed [${\text{SO}}_{4}^{2-}$] (10 μm, Figure 2) and highest observed to theoretical maximum [∑H_2_S] generation (Figure 4) occurred.

Interestingly, seven candidate divisions were identified across the 5 depths, which have individually been found across diverse habitats: (1) organic and/or sulfate rich–OP1, subsurface sediments of Guaymas Basin (Vigneron et al., 2014), (2) extreme–OP9, hypersaline microbialite forming mats in a hypersaline lake (Schneider et al., 2013) and GN02, Guerrero Negro hypersaline microbial mats (Ley et al., 2006), NKB19 deep marine (>3000 m) sediments (Li et al., 1999), as well as (3) benign–i.e., soils, Spam (Lipson and Schmidt, 2004), BRC1, municipal anaerobic sludge digestor (Chouari et al., 2005) and northern wetland peat layers (Serkebaeva et al., 2013) and (4) wide ranging occurrence–OP8, including high abundance in hydrocarbon impacted environments (Farag et al., 2014). The constellation of so many candidate divisions within CT, i.e., organisms we know the least about, highlights the importance of investigation of mine waste contexts to further our understanding of global microbial biogeography and sulfur biogeochemical cycling in a context similar, yet distinct from important SRB marine, wetlands and sedimentary contexts. Relative evolutionary rates (rERs) have been shown to be faster in extreme environments (Li et al., 2014a,b) indicating the importance of environmental conditions in shaping microbial communities and the opportunities to investigate broader ecological questions within these manmade, microbially focused and highly active communities.

### CT depth dependent sulfur and iron biogeochemical trends

Porewater geochemistry (Figure 2) identified three zones over the 34 m depth sampled: (1) active Fe^(III)^ reduction (2–4 m); (2) a transition zone where both Fe^(II)^ and ∑H_2_S were detected in porewaters (6–8 m); and (3) elevated SRB activity toward the bottom of the deposit (>22 m). Coherent microbial community phylum level, depth-dependent trends consistent with these zones also emerged (Figure 5D). Ubiquitous presence of cosmopolitan phyla (Actinobacteria, Bacterioidetes, Chloroflexi, Firmicutes, Proteobacteria, and candidate division *OP1*), contrasted: (1) candidate divisions NKB19 and Spam present only at the transition zone depth where both ∑H_2_S and Fe^(II)^ occurred (6–8 m), (2) Planctomycetes, Deinocococcus-Thermus, and Pseudoanabaena present at the transition (6–8 m) zone and the high ∑H_2_S depth (22–24 m), (3) Chlorobi, Synergistes, and candidate division BRC1 present exclusively at the depth of highest ∑H_2_S (22–24 m), and (4) Armatimonadetes, Spirochaetes, and candidate divisions OP8 and OP9 present only at the two deepest depths (22–24 and 32–34 m) associated with the greatest extent of sulfur transformation (Figure 5D).

#### Shallow Fe reduction zone (2–4 m)

The highest level of Fe^(II)^ was detected in porewaters at 2–4 m (38.5 μM) consistent with IRB activity. Concurrently, the observations of the highest ${\text{SO}}_{4}^{2-}$ concentration combined with no detectable ∑H_2_S (Figure 3), indicated that SRB are not appreciably active at this depth. This depth exhibited the lowest overall community diversity based on sequencing data, with only 5 phyla represented (Figure 5). The predominance of IRB within the 2–4 m community was supported by higher proportions of the monoenoic PLFA (Figure 6B) known to be produced by Proteobacteria, particularly 16:1Δ11 which has been identified in iron-reducing members of the genera *Geobacter* and *Shewanella*, (Lovley et al., 1990; Rooney-Varga et al., 1999) members of δ and γ Proteobacteria, classes also detected here. The highest Gamma proteobacteria abundance (71%), predominantly *Pseudomonas*, a metabolically diverse, including Fe cycling, genus (Cummings et al., 2010), was observed at this depth (Figure 5B).

#### Transition IRB-SRB activity zone (6 – 8 m)

Both Fe^(II)^ and ∑H_2_S were detected within 6–8 m porewater (Figure 2) consistent with a transition from IRB to SRB activity, and concurrent with the observation of the greatest microbial diversity (Figure 5). Interestingly, three candidate divisions GN02, NKB19 and Spam as well as ϵ Proteobacteria (*Sulfurovum*) were only observed within this 6–8 m transition zone (Figure 5D). In addition, the greatest proportion of known SRB strains were also observed at this depth (Figures 5A,E). These SRB can contribute to observed increases in both monoenoic PLFA and terminally branched PLFA observed at this depth. Desulfobacteraceae present only at this depth, are known to be active in the oxidation of short and long chain alkanes (Kleindienst et al., 2014).

The continued observation of high concentrations of the 16:1Δ11 PLFA (Figure 6B) at 6–8 m is consistent with continued abundance of IRBs. This apparent restriction of IRB activity to the first two sampling depths based on detectable porewater Fe^(II)^ (Figure 2) is interesting given the increasing concentrations of putative Fe^(III)^ substrates and TOC with depth (Table 1), suggesting IRB should not be substrate limited. We can only speculate as to why IRB activity is potentially inhibited at deeper depths. It may be that at deeper (i.e., older) depths, the bulk TOC and/or mineral Fe^(III)^ substrates remaining are too recalcitrant and thus inaccessible (i.e., Esteve-Núñez et al., 2005) and/or nutrient limiting for IRB activity. The presence of *Sulfurovum*, a known sulfur oxidizer (Wright et al., 2013) only at the apparent IRB-SRB transition depth also suggests that sulfur oxidation may also be occurring at this depth. Interestingly, Stasik et al. (2014) identified high rates of thiosulfate oxidation within an oil sands tailings pond and identified up to 10^5^–10^6^ cells /mL of sulfur oxidizing bacteria at depth within the tailings pond consistent with sulfur redox cycling. Here, the highest abundance of *Clostridium*, a thiosulfate and/or S^0^ reducer, occurred at the two shallower 2–4 and 6–8 m depths, suggesting sulfur cycling may be occurring in the surface layer of the CT deposit.

#### Deep, elevated SRB activity zone (22–34 m)

The highest relative SRB activity determined as observed ∑H_2_S:maximum theoretical ∑H_2_S possible (Equation 1, Figure 4) occurred at the two deepest depths sampled. While this is a highly simplified approach presuming all DOC was accessible, values for the two deepest depths, 67% (22–24 m) and 230% (32–34 m), identified greater ${\text{SO}}_{4}^{2-}$ transformation than those observed for the shallower depths (<2%), consistent with higher SRB activity. The high proportions of terminal branched PLFA at these depths are consistent with the presence of SRB (Taylor and Parkes, 1983). In particular a preference for *iso*- over *anteiso*- as observed at depths of 22–24 and 32–34 m depth (ca 3:1; highest proportion at 22–24 m) has been observed in members of the genus *Desulfovibrio* identified to occur at 22–24 m (Figure 5E; Taylor and Parkes, 1983; Kohring et al., 1994). Interestingly, 10me16:0 has also been detected in an anaerobic, organo-halide respiring member of the phylum Chloroflexi (Loffler et al., 2013). The quantifiable detection of this PLFA at 32–34 m may represent some contribution from related organisms and is consistent with the observed increase in Chloroflexi 16S rRNA sequences at depth. Overall, the shift in PLFA profile toward decreased 16:1Δ11 and increased i/a15:0 with depth is consistent with a relative decrease in iron-reducing bacteria and an increase in SRB biomass consistent with the observed geochemical porewater trends (Figures 2, 6).

### CT: Evidence for distinct anthropocene sulfur contexts

While of anthropogenic origin, CT bulk geochemistry was not unlike many natural sulfur and organic carbon rich environments (e.g., marine, wetland, etc.). The bulk organic carbon content of CT (0.8–1.2%; Table 1) was similar to those of organic-rich environments such as coastal marine sediments (~0.5–1.2% w/w) and artificial and natural wetlands (~1.1–2.5% w/w; Zhu et al., 2012; Peralta et al., 2013). Similarly, porewater [Fe^(II)^] (1.2–38.5 μM; Figure 2) was comparable to those of Arctic marine sediments (20–47 μM; Algora et al., 2013) and peatlands (5–150 μM; Blodau et al., 2007) but considerably higher than those reported for oil sands tailings (0.36–10.9 μM; Penner and Foght, 2010). However, despite lower diversity and abundance relative to other sulfur and organic carbon rich contexts, CT microbial communities are highly effective at sulfur redox cycling of these materials. CT background levels of porewater [∑H_2_S] (~20 μM), were substantially higher than ∑H_2_S values reported for tidally reflooded wetlands and peatlands (<2–9 μM; Blodau et al., 2007; Burton et al., 2011). Further, the highest ∑H_2_S concentration of 301.5 μM observed at 22–24 m, was within the range of environments reported to house the high levels of SRB activity such as FFT microcosm experiments amended with ${\text{SO}}_{4}^{2-}$ (400 μM; Salloum et al., 2002), Black sea sediments (0.7–435 μM; Zopfi et al., 2012), some meromictic lakes (60–530 μM; Del Don et al., 2001) and brackish coastal lake sediments (ranged from 3 to 1380 μM; Sakai et al., 2013). These results suggest that CT materials, while comparable in terms of sulfate and organic carbon concentrations to many natural environments, are differentiated by modestly diverse, low abundance, but highly sulfur active, more enigmatic communities (7 candidate divisions present within the 19 phyla identified).

## Author contributions

LW contributions: responsible for original research conception, results generation, interpretation, and primary writer of manuscript. KK contributions: responsible for sample collection, analyses, results generation with LW, and major contributions to manuscript generation. AB contributions: responsible for generation of lipid results, interpretation, and summary of these results within the manuscript. GS contributions: Co-I on original research project with LW; here, responsible for lipids results generation with AB, interpretation and written contribution to the manuscript on these results. The manuscript was written through contributions of all authors. All authors have given approval to the final version of the manuscript.

### Conflict of interest statement

The authors declare that the research was conducted in the absence of any commercial or financial relationships that could be construed as a potential conflict of interest.
