# Supplementary material for: Sulfur Biogeochemistry of an Oil Sands Composite Tailings Deposit
Source: Front Microbiol. 2016 Feb 3;6:1533. doi: 10.3389/fmicb.2015.01533 (PMC4737920; doi:10.3389/fmicb.2015.01533)
Supplement: Supplementary file 1 [file Table1.DOCX]

| Table S1 Mineralogical characterization of CT by depth by XRD | | | | | | |
| --- | --- | --- | --- | --- | --- | --- |
| Mineral | Depth of CT | | | | | Notes |
|  | 2-4m | 6-8m | 14-16m | 22-24m | 32-34m |  |
| Quartz | 61% | 56% | 52% | 43% | 44% | SiO2 |
| Calcite | 2% | 1% | 2% | 2% | 2% | CaCO3 |
| Albite/anorthite | 1% | 3% | 3% | 4% | 3% | NiAlSi3O8/CaAl2Si2O8 |
| Microcline | 4% | 5% | 6% | 5% | 4% | KAlSi3O8 |
| Dolomite (ankerite) | 4% | 2% | 4% | 3% | 3% | (CaMg)(CO3)2 |
| Goerthite | 1% | 2% | 1% | 1% | 2% | Fe hydroxide |
| Magnetite | 2% | 3% | 3% | 2% | 3% | Fe3O4 |
| Illite/micas | 8% | 9% | 9% | 14% | 14% | (KH2O)(Al, Mg, Fe)2(Si, Al)/(K,Na,Ca)2(Al,Mg,Fe)4-6(Si,Al)8O20(OH,F)4 |
| Chlorite | 2% | 3% | 3% | 4% | 4% | ClOx |
| Halite | 1% | 1% | 1% | 2% | 2% | NaCl |
| Siderite | 2% | 2% | 2% | 2% | 2% | FeCO3 |
| Kaolinite/serpentine | 8% | 9% | 10% | 13% | 12% | Al2Si2O5(OH)4/(Mg,Fe)Si2O4(OH4) |
| Amorphous, ect. | 4% | 4% | 4% | 5% | 5% |  |
| SUM | 100% | 100% | 100% | 100% | 100% |  |
| Fe Sum | 25% | 29% | 29% | 37% | 38% |  |
