# Supplementary material for: Sulfur Biogeochemistry of an Oil Sands Composite Tailings Deposit
Source: Front Microbiol. 2016 Feb 3;6:1533. doi: 10.3389/fmicb.2015.01533 (PMC4737920; doi:10.3389/fmicb.2015.01533)
Supplement: Supplementary file 2 [file Table2.DOCX]

Table S2. Drill water (unavoidable contamination) sequences removed from CT depth sample sequence datasets and subsequent depth dependent community characterization. Data shown provides the % that the OTU (organized by phylum and class) represents of the clone library for any given depth as well as the raw count of sequences in parentheses ().

|  | No. of OTUs | % of clone library (no. of clones) | | | | | | | | | | |  |
| --- | --- | --- | --- | --- | --- | --- | --- | --- | --- | --- | --- | --- | --- |
|  |  | 2-4 m | | 4-6 m | | 14-16 m | | 22-24 m | | 32-34 m | |  | |
| Acidobacteria |  |  |  |  |  |  |  |  |  |  |  |  |  |
| Acidimicrobia | 1 |  |  |  |  |  |  |  |  |  |  |  |  |
| Actinobacteria | 9 | 0.6% | (19) | 1% | (91) | 0.3% | (14) | 0.6% | (53) | 0.3% | (16) |  |  |
| Coriobacteria | 1 | 0.2% | (6) | 0.2% | (16) |  |  | 0.1% | (8) | 0.1% | (3) |  |  |
| Holophagae | 1 |  |  |  |  |  |  |  |  |  |  |  |  |
| Bacteroidetes |  |  |  |  |  |  |  |  |  |  |  |  |  |
| Flavobacteria | 4 |  |  | 0.30% | (20) |  |  | 0.20% | (14) |  |  |  |  |
| Sphingobacteria | 1 | 0.20% | (7) |  |  |  |  |  |  |  |  |  |  |
| Bacteroidia | 1 |  |  |  |  | 0.20% | (9) |  |  |  |  |  |  |
| BRC1 | 1 |  |  |  |  |  |  |  |  |  |  |  |  |
| Chlorobi |  |  |  |  |  |  |  |  |  |  |  |  |  |
| Ignavibacteria | 1 |  |  |  |  |  |  | 0.10% | (6) |  |  |  |  |
| Chloroflexi |  |  |  |  |  |  |  |  |  |  |  |  |  |
| Dehalococcoidetes | 1 |  |  |  |  | 0.10% | (5) |  |  |  |  |  |  |
| Anaerolineae | 3 | 0.40% | 13 | 0.40% | 31 | 0.10% | 6 | 3% | 232 | 6% | 286 |  |  |
| Firmicutes |  |  |  |  |  |  |  |  |  |  |  |  |  |
| Bacilli | 15 | 0.30% | (10) | 1% | (82) | 1% | (49) | 0.70% | (69) | 0.70% | (33) |  |  |
| Clostridia | 21 | 0.10% | (2) | 5% | (367) | 0.30% | (13) | 2% | (150) | 0.40% | (20) |  |  |
